# Human core duplicon gene families: game changers or game players?

**DOI:** 10.1093/bfgp/elz016

**Published:** 2019-09-16

**Authors:** Cemalettin Bekpen, Diethard Tautz

**Affiliations:** Max-Planck Institute for Evolutionary Biology, 24306 Plön, Germany

**Keywords:** duplication, duplicons, gene family, human, adaptation, copy number variation

## Abstract

Illuminating the role of specific gene duplications within the human lineage can provide insights into human-specific adaptations. The so-called human core duplicon gene families have received particular attention in this respect, due to special features, such as expansion along single chromosomes, newly acquired protein domains and signatures of positive selection. Here, we summarize the data available for 10 such families and include some new analyses. A picture emerges that suggests broad functions for these protein families, possibly through modification of core cellular pathways. Still, more dedicated studies are required to elucidate the function of core-duplicons gene families and how they have shaped adaptations and evolution of humans.

## Introduction

**Figure 1:**
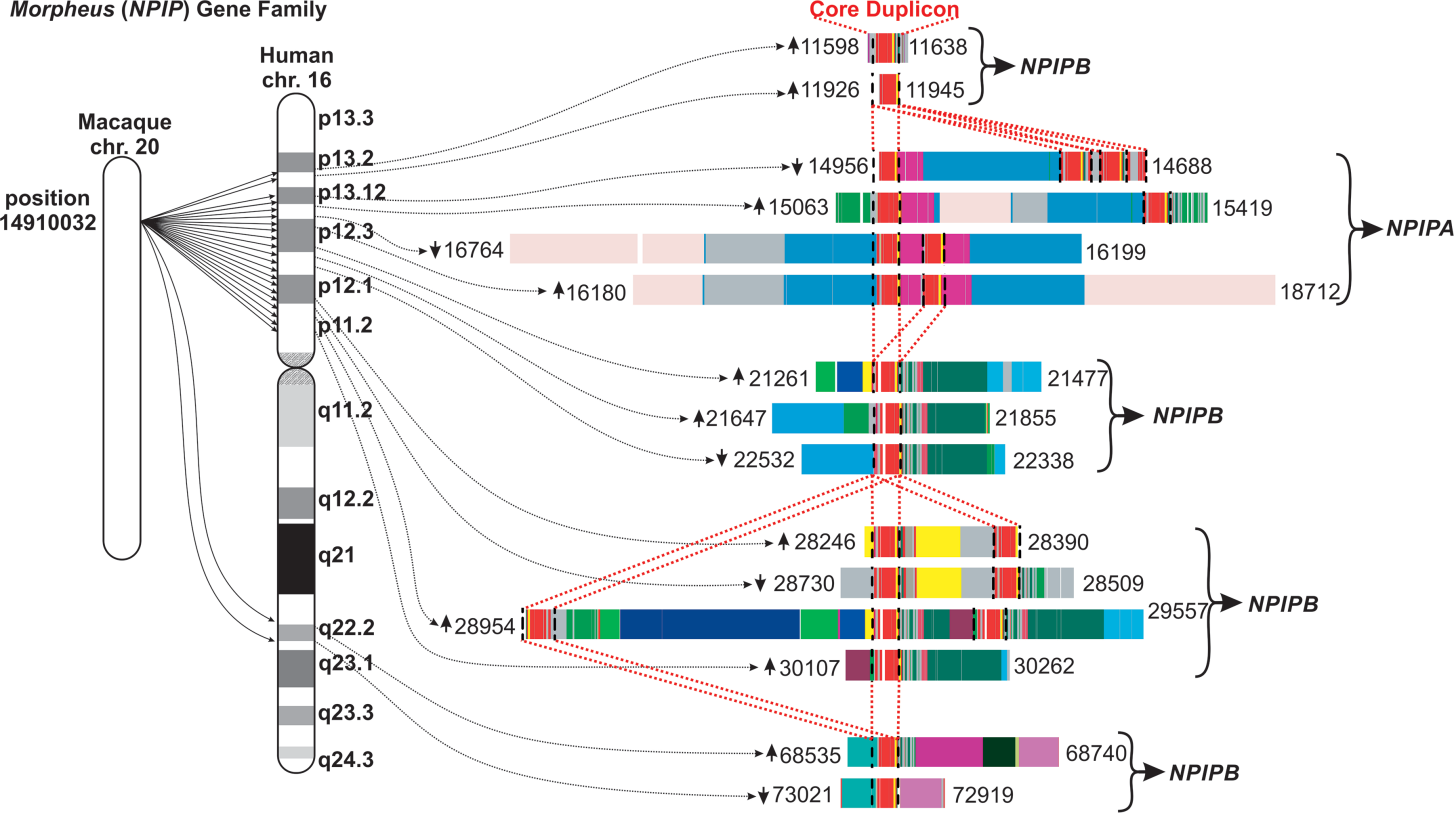
The *Morpheus* (*NPIP*) gene family as an example for human core duplicons. Figure adapted from [4, 7]. The individual members of the *Morpheus* core duplicons are ordered according to location on chromosome 16 (left). The mosaic structure of the complex duplication blocks is depicted on the right. The core duplicon part, which is shared by all duplication blocks, is marked by the red broken lines. Numbers next to the fragments refer to the start and end positions on the chromosome (in kb), and arrows indicate the relative orientation. Different colors refer to distinct duplication blocks, which are predicted based on reciprocal comparisons of each human subunit and its flanking sequence to outgroup mammalian genomes [4].

The human genome harbors a number of rapidly evolving gene families that have been subjected to a combination of structural reorganization and bursts of segmental duplications between one to several hundred kilobases. Approximately 400 blocks of the human genome have been identified as having undergone multiple duplications during hominoid evolution. Overall, segmental duplications comprise approximately 5% of the human genome [1–3]. A detailed analysis of these segmentally duplicated regions has shown that subsets are formed around ‘core’ or ‘seed’ duplicons that are shared between all copies of the respective gene family [4–6]. Figure 1 shows the duplication structures of the *Morpheus* (*NPIP*) gene family as an example, which will be discussed in further detail below.

We focus here on 10 core duplicon gene families, which share a number of other features. (i) Their duplicates are generally confined to a single chromosome, partly in tandem but also dispersed along the chromosome. (ii) They have variable copy numbers in human populations and include some of the most variable human CNV genes (e.g. *SPATA31, Morpheus (NPIP)* and *LRRC37A*) [8–11]. (iii) Almost all of these genes show ubiquitous or at least broad patterns of expression, while their ancestral progenitor genes often exhibit tissue-specific expression, mostly in the testis [4]. Intriguingly, at least half of these core duplicon gene families (including *Morpheus (NPIP)*, *SPATA31*, *NBPF11, RGPD, GOLGA, PMS2P and TRIM51*) evolved under positive selection (Table 1), making them among the fastest evolving genes in humans [12–17]. Hence, both the human lineage-specific expansion and the patterns of positive selection suggest that these gene families have played a direct role in the phenotypic evolution of humans.

**Table 1:** Signatures of positive selection in human core duplicons

Chr.	Name	Alternative name(s)	Ancestral gene (mouse)	Signatures of positive selection	Selection test	Literature
1	Olduvai protein domain family	Neuroblastoma breakpoint gene family, NBPF	Pde4dip	Yes	PAML, *dN/dS*, The likelihood ratio test, *Ka/Ks,* Nei-Gojobori	[13,16]
2	RGPD	RANBP2-like gene family	Ranbp2	Yes	PAML, *dN/dS*, The likelihood ratio test, *Ka/Ks,* Nei-Gojobori	[13,15]
5	SMA-GUSBP	SMA	Gusbp2	None described		
7	PMS2P	PMS2 gene family	Pms2	Yes	PAML, *dN/dS*, The likelihood ratio test,	[12]
9	SPATA31	FAM75A	Spata31	Yes	PAML, *dN/dS*, The likelihood ratio test,	[13]
11	TRIM51	SPRYD5	no homolog	Yes	PAML, *dN/dS*, The likelihood ratio test,	[17]
15	GOLGA8	GOLGA, golgin	Golga2	Yes	PAML, *dN/dS*, The likelihood ratio test,	[12,13]
16	Morpheus	NPIP	no homolog	Yes	PAML, *dN/dS*, The likelihood ratio test, *Ka/Ks,* Nei-Gojobori	[13,14]
17	TBC1D3	-	Usp6nl	None described		
17	LRRC37	-	Lrrc37a1	None described		

**Figure 2:**
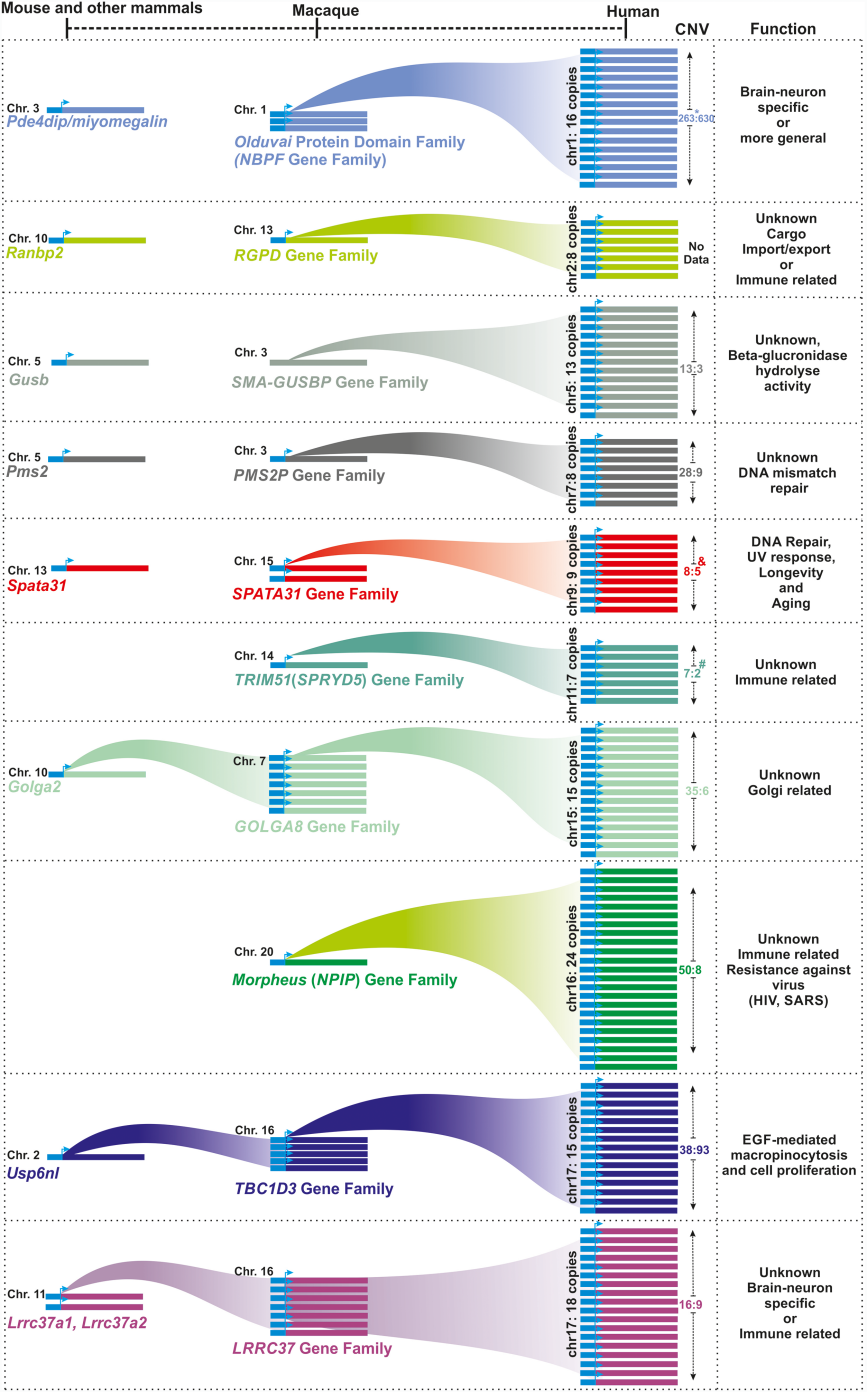
Overview of human core duplicon gene families. The figure depicts the segmentally duplicated copies of core duplicon gene families from human, macaque and mouse and includes other mammals. Functional properties of *Olduvai*, *SPATA31* and *TBC1D3*, which have been studied in detail, and possible/predicted functions of other core duplicon gene families are included. CNV (median:variance) was extracted from a previous study [10]. * indicates CNV estimate based on number of Olduvai domains. ^#^ indicates CNV information for *TRIM51* genes was extracted from a previous study [17], and ^&^ indicates CNV for *SPATA31* genes was extracted from a previous study [18].

Since the initial report of human core duplicons [4], most of the genes belonging to duplicons were evolutionarily and structurally characterized. However, only three (*NBPF*, *TBC1D3* and *SPATA31*) were studied functionally in more detail. Here, we provide an overview on our current knowledge on the evolution and function of gene families that are parts of the human core duplicons. We describe these gene families according to their order along the human chromosomes. The general overview is provided in Figure 2.

### Chromosome 1: *Olduvai* protein domain (*NBPF* gene) family

#### Evolution and comparative genomics

The initial name ‘Neuroblastoma Breakpoint gene Family’ *(NBPF)* was given because the first identified member of the family was found to be deleted in an individual with neuroblastoma [19]. *NBPF* gene family members include variable numbers of tandemly repeated DUF1220 domains within their coding sequences. Since the name DUF1220 was initially a working designation assigned by the Pfam database curators, Sikela and van Roy [20] have proposed to rename the domain Olduvai. The different *NBPF* core duplicon genes are distributed along chromosome 1 in 16 copies, of which 6 are in tandem and 10 are dispersed [21]. The macaque genome has three copies of *NBPF* genes, while mice and other mammals have no clear orthologs. A possible ancestral gene is *PDE4DIP*, which includes a single Olduvai domain [22]. The Olduvai domain copy number has particularly expanded in humans. It increased from a single domain in mouse to 30–35 copies in new and old world monkeys and up to 300 domains in humans [22]. Based on sequence similarity, the domain structure of Olduvai can be divided into six primary subtypes including CON 1, 2 and 3 and HLS 1, 2 and 3 [16, 22, 23]. The comparative analysis of Olduvai-domain-containing *NBPF* genes in primates has shown that *NBPF* genes evolve under strong positive selection [13, 16].

#### Expression and subcellular localization

*NBPF* genes are ubiquitously expressed in all human tissues and exhibit elevated expression in the testis. The promoter region of *NBPF1* genes is derived from the unrelated gene *EVI5* [22, 24]. Over-expressed, myc-tagged NPBPF1 protein in human breast carcinoma cells (MCF7) was shown to be primarily localized to the cytoplasm [23].

#### Function

Despite its broad expression, the functional analysis of this gene family has focused on brain- and/or neuron-specific functions. This was triggered by the observation that the 1q21.1 chromosome region in humans, which is associated with brain disorders such as micro- or macrocephaly, autism and schizophrenia, includes a number of the *NPBPF1* genes [25]. Further, a correlation was found between Olduvai copy number and brain size in humans. This correlation was most prominent in affected patients, but copy number was also associated with gray-matter volume in healthy controls [26]. Over-expression of Olduvai sequences in neural stem cells promotes proliferation [27–29]. Further studies also suggested a correlation between Olduvai copy number and schizophrenia risk and severity [30] as well as with IQ and total mathematical aptitude scores [31], although the breadth of this latter study is limited. While these results suggest a particularly interesting function of *NBPF* genes in human brain evolution with implications for the etiology of brain diseases [21], more studies are required to support this notion. Notably, copy number variations (CNVs) and deletions in the 1q21.1 region are also correlated with heart [32, 33] and kidney [34] diseases. Given that *NBPF11* genes are broadly expressed, they could have a more general function. In a yeast two-hybrid system, *NBPF11* was shown to interact with *Chibby,* which is a negative regulator of the *Wnt* signaling pathway [35]. There are currently no data supporting a possible specific molecular function for Olduvai domains in humans. In mouse, targeted deletion of the Olduvai domain in the *Pde4dip* gene resulted in not only significantly reduced fecundity and hyperactivity of mice but also in physiological changes and liver function-related effects [36].

#### Correlation with brain size

As discussed above, there is a strong emphasis in the literature that *NBPF/Olduvai* copy numbers are correlated with increasing brain size in primates and humans. However, given that the pattern of gene family expansion also holds for the other core duplicon gene families discussed here, we have re-assessed whether the same correlation is more broadly apparent. Using the brain volume measures previously provided [29] and the number of currently annotated gene copies for the various families in different genomes, we found that all these gene families show a significant correlation. In contrast, there is only a partial correlation with body mass (Table 2). Hence, one can either conclude that all human core duplicon gene families influence brain size or the correlation is incidental because these gene families are of increased interest due to their special expansion in humans.

**Table 2:** Linear regression analysis of human core duplicon gene families with brain volumes and body mass across primates

	NBPF / Olduvai	RGPD	SMA-GUSB	PMS2P	SPATA31	TRIM51 (SPRYD5)	GOLGA	MORPHEUS	TBC1D3	LRRC37
Brain mass (mg)	0.90^**^	0.91^**^	0.90^**^	0.38^*^	0.72^**^	0.87^**^	0.68^**^	0.66^**^	0.82^**^	0.83^**^
Brain volume (mm^3^)	0.90^**^	0.91^**^	0.90^**^	0.38^*^	0.72^**^	0.87^**^	0.68^**^	0.66^**^	0.83^**^	0.83^**^
Neocortex volume (mm^3^)	0.92^**^	0.90^**^	0.92^**^	0.37^*^	0.72^**^	0.86^**^	0.68^**^	0.65^**^	0.81^**^	0.83^**^
Cerebellum volume (mm^3^)	0.83^**^	0.91^**^	0.81^**^	0.36^*^	0.72^**^	0.85^**^	0.64^**^	0.64^**^	0.81^**^	0.77^**^
Body mass (g)	0.23^ns^	0.57^**^	0.24^ns^	0.22^ns^	0.38^*^	0.43^*^	0.28 ^ns^	0.35^*^	0.44^*^	0.27 ^ns^

^*^ *P* < 0.05.

^**^ *P* < 0.001.

^ns^ *P* > 0.05.

### Chromosome 2: RANBP2 (RGPD) gene family

#### Evolution and comparative genomics

*RGPD genes* (RanBP2-like, GRIP domain-containing Proteins) are derived from the *RANBP2* gene. *RANBP2* (*NUP388*) is a Ran-binding protein that was shown to interact with the nuclear pore complex. The *RGPD* gene family has expanded to eight dispersed copies on human chromosome 2 through segmental duplication [15]. Similar to other core duplicon gene families, *RGPD* genes are rapidly evolving. Different exons of *RGPD* genes were shown to evolve under positive selection [15]. The macaque genome, as well as the mouse and other mammalian genomes, does not contain duplicates, suggesting that expansion occurred within the great apes after the separation from old world monkeys.

#### Expression and subcellular localization

*RGPD* genes are ubiquitously expressed in human tissue with elevated expression in the testis [15]. Over-expression of GFP-fused *RGPD5-7* revealed its localization to the cytoplasm around the nuclear envelope [15, 37].

#### Function

The RANBP2 protein encoded by the progenitor gene is primarily localized within the periphery of the nuclear envelope and is thought to be required for cargo import and export [37]. Hence, the *RGPD* gene family members may be modifiers of this function. Interestingly, *RANBP2* was also shown to be involved in resistance against Simian Immunodeficiency Virus [38]. It is thus possible that the expansion of *RGPD* genes is the result of an arms race between virus evolution and host resistance acquisition. The *Ranbp2* knockout in mice is homozygous lethal.

### Chromosome 5: SMA (GUSBP) gene family

#### Evolution and comparative genomics

The repetitive nature of the spinal muscular atrophy (*SMA)* genes was first described [39] in the context of searching for candidate genes for SMA. However, SMA is caused by mutations in the duplicated copies of the *SMN1* and *SMN2* genes, which are located at chromosome 5q13.3 (reviewed in detail in [40]). *SMA* core duplicon copies are located in close proximity both upstream and downstream (within approximately 50 kb) of the *SMN2* gene. Therefore, to avoid confusion with the disease-causing genes, we will use the designation *SMA-GUSBP* to represent the *SMA* gene family***.** SMA-GUSBP* genes are organized in 2 tandem and 10 dispersed copies along human chromosome 5; an additional copy is located on chromosome 6, and this is most slikely the ancestral copy. In the common ancestor of chimp and human, the ERV1/LTR12C retroviral element integrated upstream of *SMA-GUSBP*. In macaque, the *GUSBP* and *ERV1*/LTR12C elements are found in different chromosomal locations (Supplementary Fig. 1). The *SMA-GUSBP* gene encodes a beta-glucuronidase-like domain (Supplementary Fig. 2). No homologs of the *SMA-GUSBP* genes have been observed outside the great apes to date, i.e. it expanded specifically in the human lineage [10]. Therefore, *SMA-GUSBP* genes are the youngest expanded core duplicon family in humans.

#### Expression and subcellular localization

The human *SMA-GUSBP* mRNA transcripts include different splicing isoforms that extend more than 70 kb. The genes are broadly expressed, and the highest expression (15–20 fold) has been detected in the testis, thymus, brain and cerebellum (Supplementary Fig. 3A-B). Transiently over-expressed, C-terminally FLAG-tagged *SMA-GUSBP* proteins localize primarily in the vesicles of HeLa cells (Supplementary Fig. 3C).

#### Function

Although *SMA-GUSBP* genes were initially thought to be associated with SMA disease, there is no current evidence to support this, i.e. the close proximity of *SMA-GUSBP* genes to the *SMN2* gene may not imply a functional connection. No direct functional analysis of *SMA-GUSBP* genes has been conducted so far.

### Chromosome 7: PMS2P gene family

#### Evolution and comparative genomics

The *PMS2P* gene family is derived from the *PMS2* gene, which encodes a homolog of the *mutL* mismatch repair gene from bacteria [41]. *PMS2P* genes duplicated to eight dispersed copies (*PMS2P1–5, 7, 9* and *11*) from the C-terminal region of the *PMS2* gene located on human chromosome 7 [42]. The current marmoset and macaque genome assemblies do not include an intact *PMS2P* gene. However, the Orangutan genome includes three complete *PMS2P* copies. This suggests an expansion of *PMS2P* genes occurred within great apes. More work will be required to show whether they were lost in some primate genomes, or whether this is still an annotation problem. The *PMS2P* gene region is highly repetitive and contains multiple transposable elements (SINEs and LINEs), which make its annotation problematic.

#### Expression and subcellular localization

Similar to other core duplicons, *PMS2P* genes are expressed ubiquitously and have enhanced expression in the testis (as annotated in the NCBI description). No further data is available on the subcellular localization of these proteins.

#### Function

No functional analysis has been performed for *PMS2P* genes to date.

### Chromosome 9: SPATA31 gene family

#### Evolution and comparative genomics

*SPATA31* (formerly known as *FAM75A*) genes expanded from a single copy in mouse to two copies in macaque and at least nine copies on human chromosome 9 by segmental duplication. Seven of these duplicated *SPATA31* gene segments are distributed on the long arm, and at least two are in tandem on the short arm. Two copies on the long arm are considered pseudogenes due to premature stop codons [18]. During the process of duplication within the primate lineage, the 5′-region of *SPATA31* genes acquired an additional exon and additional protein domains, in particular a cryptochrome domain [18]. Similar to other core duplicon gene families, the *SPATA31* promoter region became highly restructured before or during the duplication events. This restructuring includes the integration of a transposable element (LINE/L1, PA10-12) after the split between simians and prosimians [18].

#### Expression and subcellular localization

*SPATA31* gene expression evolved from testis-specific expression in mice and macaques to ubiquitous expression in humans, but it still exhibits its highest expression in the testis [18]. Endogenous SPATA31 protein shows a dynamic localization between the cytoplasm and nucleus depending on fixation conditions and exposure to light [18]. In cell culture, SPATA31 protein re-localizes from the nucleolus to the nucleus upon UV exposure [18].

#### Function

*SPATA31’s* UV-exposure response, as well as its acquired protein domains, suggests a function in DNA damage repair. Functional analysis of cells with reduced *SPATA31* copy number showed increased sensitivity to UV exposure [18]. Furthermore, over-expression of *SPATA31A1* in epidermal cells leads to premature senescence [43]. Interestingly, long-lived individuals (>96 years old) have significantly fewer copies of *SPATA31* genes on average compared to a control group. This observation suggests that the adaptive evolution of the *SPATA31* gene family is an example of antagonistic pleiotropy; it provides a fitness benefit during the reproductive phase of life (better protection against UV-light damage), but it negatively influences overall life span possibly by causing more repair-induced mutations [43]. Knockout of the progenitor gene in mice leads to spermatogenesis defects and infertility [44], which is in line with its testis-specific expression in mice.

### Chromosome 11: TRIM51 (SPRYD5) gene family

#### Evolution and comparative genomics

A first description of the evolution of *TRIM51* genes is included in a general study of *TRIM* genes by Han et al. [17], who showed that *TRIM51* genes are recently duplicated, hominoid-specific *TRIM* genes. Some of the *TRIM51* genes (C1,C2) were found to have CNV in human individuals and to evolve under positive selection [17]. The human genome assembly includes seven full-length, duplicated copies of *TRIM51 (SPRYD5)* genes, at least five fragmented duplication blocks that are expanded mainly within the centromeric region of chromosome 11 and an additional copy on chromosome 2 (*TRIM51JP*). Comparisons of the available assembled genomes (rheMac8, ponAbe3 and hg38) in the UCSC genome browser and Synteny in Ensembl indicate that *TRIM51* genes initially evolved by segmental duplication from *TRIM51EP*. There is a single full-length copy in the macaque genome, which shows high similarity to human *TRIM51EP*. However, more detailed work is required to resolve this.

#### Expression and subcellular localization

Low level *TRIM51* expression was detected in the developing brain and testis (https://www.ebi.ac.uk/gxa/home). Although classified as *TRIM* genes, members of the *TRIM51* gene family lack several important motifs and domains that are otherwise common to TRIM proteins (e.g. RING and B-Box 2) [45]. TRIM51 proteins contain a coiled-coil domain and a SPRY domain. To date, no reports have shown the subcellular localization of TRIM51 proteins.

#### Function

In general, TRIM- or SPRYD-domain-containing genes are involved in functions related to innate immune response [17, 46]. However, there have been no studies investigating the function of TRIM51 proteins as an expanded gene family in humans because they are not classified with other *TRIM-* or *SPRYD*-containing genes [45]. But a possible involvement in immune response mechanisms could evidently explain their adaptive evolution.

### Chromosome 15: GOLGA gene family

#### Evolution and comparative genomics

*GOLGA* (*GOLGIN*) genes encode long coiled-coil proteins associated with the Golgi apparatus. They form a large gene family distributed across different chromosomes in humans. Several of the *GOLGA* genes are ancient duplicates with orthologous copies in the mouse (*GOLGA1*, *GOLGA2* on chr9, *GOLGA3* on chr12, *GOLGA4* on chr3, *GOLGA5* on chr14, *GOLGA7* on chr8 and *GOLGA7B* on chr10). However, there are also two subfamilies—*GOLGA6* and *GOLGA8—*that share similarity to each other. GOLGA8 has human-specific core duplicons that are expanded along chromosome 15 by segmental duplications and are partially in tandem. The current human assembly contains 12 annotated duplicated *GOLGA6* subfamily members, whereas the *GOLGA8* subfamily includes 15 duplicated copies. They show the closest similarity to *GM130* (*GOLGA2*), which is located on human chromosome 9 [47]. The macaque genome contains approximately 11 *GOLGA6*-like and at least 8 fragmented *GOLGA8*-like duplicates. The N-terminal portions of *GOLGA8* genes show high variation in both macaque and human. The duplication architecture for *GOLGA6* and *GOLGA8* copies is complex and not yet well resolved.

#### Expression and subcellular localization

Both *GOLGA6* and *GOLGA8* are ubiquitously expressed at low levels in human tissue, and they are most highly expressed in the testis. There is no direct evidence for the subcellular localization of GOLGA6 and GOLGA8 proteins; however, based on similarity to GM130, they are predicted to localize to the Golgi apparatus, Golgi stack membrane and cytoplasm [47].

#### Function

The roles of the individual variants of the GOLGA proteins are not clear [47], but they may function in membrane trafficking or Golgi structure. Loss of individual *GOLGA* genes in mice or humans is not cell lethal, possibly due to functional redundancy between different copies. Palindromic *GOLGA8* core duplicons promote recurrent chromosome 15q13.3 microdeletions that are associated with intellectual disability, schizophrenia, autism and epilepsy [48, 49] but there are currently no data that would suggest a causative role of GOLGA8 losses for the disease effects.

### Chromosome 16: Morpheus (*NPIP*) gene family

#### Evolution and comparative genomics

The ‘*Morpheus*’ gene family, also named the nuclear pore interacting protein (*NPIP*) family, is one of the best studied human core duplicon gene families. It is derived from a name-giving ‘low-copy repeat sequence on chromosome 16’, LCR16a, that is approximately 20 kb long and expanded in the great ape–human lineage along chromosome 16 through segmental duplications [7, 14, 50]. It can be subdivided into two distinct subfamilies, *NPIPA* and *NPIPB*, which mostly differ with respect to exon 5 and the structure of amino acid repeats in the C-terminus [7]. In contrast to other core duplicon gene families, it has not been possible to identify paralogs outside of primates, i.e. it appears to be a newly evolved or rapidly evolving gene. In fact, the *Morpheus* gene family was shown to be one of the most rapidly evolving gene families during hominoid evolution [14].

#### Expression and subcellular localization

*Morpheus* genes are expressed in various tissues and are most highly expressed in the testis and thymus [7]. The 5′-exons show extensive variation in splicing [7]. Due to this alternative splicing and differences in the C-terminal repeat region, Morpheus proteins vary in size between 40 and 95 kDa. Over-expression of different types of *NPIPB* and *NPIPA* genes reveals different subcellular localizations within both the nucleus and cytoplasm [7].

#### Function

Although Morpheus proteins were initially suggested to interact with the nuclear pore complex [14], there has not yet been evidence to support this assumption. They were shown to be over-expressed in the retina of patients with macular degeneration [51], but the functional significance of this is unclear. Other observations indicate an involvement in innate immunity, especially with respect to viruses. Morpheus proteins were suggested to be involved in human immunodeficiency virus resistance [52, 53], were shown to interact with Severe Acute Respiratory Syndrome Coronavirus (SARS-CoV) and were shown to restore the interferon-beta response in SARS-CoV cells [54]. Both *Morpheus* gene types (*NPIPA* and *NPIPB*) are also upregulated upon polyinosinic:polycytidylic acid (poly I:C) treatment (viral mimic), and auto-antibodies against NPIPB protein in humans have been detected [7]. It is thus possible that *Morpheus* genes could cause autoimmune diseases such as multiple sclerosis, systemic lupus erythematosus, myasthenia gravis and Wegener’s granulomatosis [55, 56]. Notably, newly evolved genes in mammals can generally create complications with respect to generating autoimmune responses [57].

### Chromosome 17: TBC1D3 gene family

#### Evolution and comparative genomics

The *TBC1D3* gene family is characterized by the TBC domain, which plays a major role in endocytosis and intracellular trafficking in other proteins. *TBC1D3* genes are derived from *USP6NL* (alias *RNTRE*) by segmental duplication [58]. Comparative genomic analysis suggests that *TBC1D3* genes emerged before the split of new world monkeys since the macaque genome (rheMac8) includes at least five copies of *TBC1D3*-like genes. *TBC1D3* genes are duplicated along human chromosome 17 in 12 copies including some processed pseudogenes [59, 60]. *TBC1D3* genes show extensive CNV between different populations (10 copies in Europeans and up to 50 copies in African populations) [10].

#### Expression and subcellular localization

*TBC1D3* genes are broadly expressed in many tissues and are most highly expressed in the testis [59, 60]. Different isoforms are expressed in different tissues [60]. In HeLa cells, ectopic expression of *TBC1D3* genes led to alterations of the actin cytoskeleton of HeLa cells. Over-expressed TBC1D3 protein localized in the cytoplasm, in lipid rafts and on the plasma membrane [58, 61, 62].

#### Function

*TBC1D3* was first described as an oncogene [63] and was also identified as *PRC17* during the analysis of cells derived from prostate and breast cancer patients [64]. A predicted GAP activity of the TBC domain is equivocal. While a weak GAP activity was documented in one study [64], this finding could not be confirmed by others; however, *TBC1D3* is still thought to be involved in macropinocytosis [58]. *TBC1D3* dysregulates the epidermal growth factor receptor signal transduction pathway and enhances cell proliferation [61]. Further studies showed that TBC1D3 protein is ubiquitinated and palmitoylated, and degradation of TBC1D3 protein is regulated by Cul7 [62, 65]. *TBC1D3* genes were also shown to be involved in Insulin/IGF signaling [66]. Over-expression of *TBC1D3* leads to an increase in cell proliferation in basal regions of the developing mouse cortex, as well as disruptions to adherens junctions and formation of column-like structures [67]. Similar results were also obtained in cultured human brain slices, where it was shown that *TBC1D3* is critical for the generation of outer radial glial cells [67]. Finally, it was shown that *TBC1D3* affects the migration of human breast cancer cells by regulating TNFα/NF-κB signaling [68].

### Chromosome 17: LRRC37 gene family

#### Evolution and comparative genomics

The *LRRC37* (leucine-rich repeat containing 37A) gene family has expanded on human chromosome 17 [4] from a single ancestral copy in other mammals [69, 70]. In humans, this family’s gene structure is highly fragmented, and only 8 of 18 copies are complete. Two of these eight complete *LRRC37* genes can be classified as the *LRRC37B* type. The number of the duplication segments is variable within the primate lineage; there are 4, 7, 10 and 18 copies in marmoset, macaque, orangutan and human, respectively [70]. Similar to the *SPATA31* genes, the N-terminal region (especially exon 1) of *LRRC37A* has acquired novel structures and promoters in the primate lineage [69].

#### Expression and subcellular localization

*LRRC37* is broadly expressed in primates, partly due to the acquisition of promotor elements from unrelated genes; however, it is still most highly expressed in the testis [69, 70]. Over-expressed LRRC37A1 protein is primarily localized to the plasma membrane. Pulse-chase experiments show that it is first localized to the Golgi and then transported to the plasma membrane where it co-localizes with Ezrin [70].

#### Function

The LRRC37 proteins contain six leucine-rich repeat motifs (LRR), which consist of repeating 20–30 amino acid stretches. Well-known LRR domain-containing proteins include those of the innate immune system, especially in mammals and plants. For example, Toll-like receptors are single-membrane-spanning proteins, and their extracellular domains are composed of LRRs, which recognize pathogen-associated molecular patterns such as LPS, single-stranded RNA and flagellin. LRR-containing proteins may also be involved in neuron-specific functions, such as axonal guidance and neuronal migration [71, 72]. However, it is not known whether *LRRC37* genes play a role in any of these functions. On the other hand, the *LRRC37* gene family in humans is located at the boundary of a common inversion polymorphism of approximately 970 kb at 17q21.31 [69, 73, 74]. This has been shown to be a significant risk factor locus for the tangle diseases, including progressive supranuclear palsy [75], corticobasal degeneration [76, 77], Parkinson’s disease [78, 79] and Alzheimer’s disease [80], and it is associated with microdeletion syndromes [81–83]. *LRRC37B*, a member of the LRRC37 gene family is also a breakpoint for NF1 microdeletion syndrome [84].

## Discussion

The evolutionary patterns of human core duplicon gene families raise a number of challenging questions. First, what is the role of the cores in the duplication process? Second, why are the non-tandem segmental duplications mostly confined to single chromosomes? Third, why are the human core duplicon genes usually derived from genes that are highly expressed in the testis? Fourth and arguably most importantly, what has the role of human core duplicon gene families been in human evolution? The data available for a number of the gene families suggest that they may not have simple single functions but instead pleiotropic effects. While it seems natural that most studies have focused on inferred specific functions, which are often related to possible disease mechanisms, we advocate for investigators to keep a broad picture in mind. For example, a number of studies have focused on possible brain expansion effects for *DUF1220/Olduvai* repeats in the lineage toward humans, but we show that such correlations can be drawn for all of the core duplicon gene families (Table 1). Our own work with *SPATA31* had initially focused on the repair of UV-induced DNA damage because of the newly acquired protein domains. However, the incidental observation that fibroblast cells with manipulated functional copy numbers of *SPATA31* exhibited altered senescence led us to study a possible connection to aging.

The most direct connections of core duplicon gene families lie in their involvement in causing chromosomal aberrations and microdeletions. But this could be due to their general repeat structure, which makes them prone to recombinational unequal cross-over mechanisms that would also affect other genes in the respective regions. Hence, the involvement in such diseases may not necessarily serve as a direct pointer toward their protein functions.

All the core duplicon gene families include expansions of protein domains, of which many are known to be involved in basic cellular functions. Hence, it seems likely that they can molecularly interact with the target genes of their progenitor proteins and thus possibly modify or regulate their core functions. Future studies should focus more on this possibility.

The duplication process included the integration of repetitive transposable elements, LTRs (e.g. ERV1) or unrelated promoters (*DND1* and *BPTF* for *LRRC37A* genes or *EVI5* for *NBPF1* genes). This integration broadened the expression to more tissues or caused ubiquitous expression. The highest expression usually remained in the testis, although the functional consequences of this expression have not been studied much to date. For example, dedicated studies should be designed to evaluate the effect of CNV of core duplicon genes on fertility phenotypes in humans.

Further, core duplicon genes commonly exist in two versions of gene structure; for example, *SPATA31A* versus *SPATA31C*, *NPIPA* versus *NPIPB*, *LRRC37A* versus *LRRC37B*, *GOLGA6* versus *GOLGA8* and *TBC1D3* (1st cluster, A–D) versus (2nd cluster, E–H). This points to diversification of effects during the duplication process, a concept that will also require more dedicated molecular studies.

Finally, it remains to be shown whether the expansion of core duplicon gene families is a specific phenomenon in the human lineage and whether they acted as ‘game changers’ with respect to human-specific adaptations. It has been possible to readily detect duplicon gene families in humans because of the abundant high-quality data and comparative genome sequencing efforts in the primate phylogeny. However, there are now also examples of lineage-specific expansions in other clades, such as rodents [85] and elephants [86]. Still, the specific duplicates in the human lineage could evidently have contributed to human-specific phenotypes and adaptations. After all, the signatures of positive selection found around human core duplicon gene families suggest an active evolutionary role. However, these signatures would likely also arise if these genes were mere modifiers of other major changes in core pathways, i.e. if they were ‘game players’. Hence, deeper studies will be required to solve the questions raised above.

**Key Points**

- Core duplicon gene families constitute a specific subset of gene duplicates in the human genome.
- They are derived from testis-expressed genes, have acquired new promoters and have specifically expanded in the evolutionary lineage toward humans.
- They express proteins with domains that suggest that they could be modifiers of general cellular pathways.
- Patterns of positive selection within the genes suggest that they were important for shaping the specific adaptations of humans.

## Biographical notes

The authors work at the Max-Planck Institute for Evolutionary Biology on patterns of genome evolution in mammals with a specific interest in newly evolved genes. D.T. is member of the editorial board of BFG.

## Supplementary Material

Supplementary_figures_elz016
