# Supplementary material for: Human core duplicon gene families: game changers or game players?
Source: Brief Funct Genomics. 2019 Sep 16;18(6):402–11. doi: 10.1093/bfgp/elz016 (PMC6920530; doi:10.1093/bfgp/elz016)
Supplement: Supplementary_figures_elz016 [file supplementary_figures_elz016.docx]

**Supplementary figures**


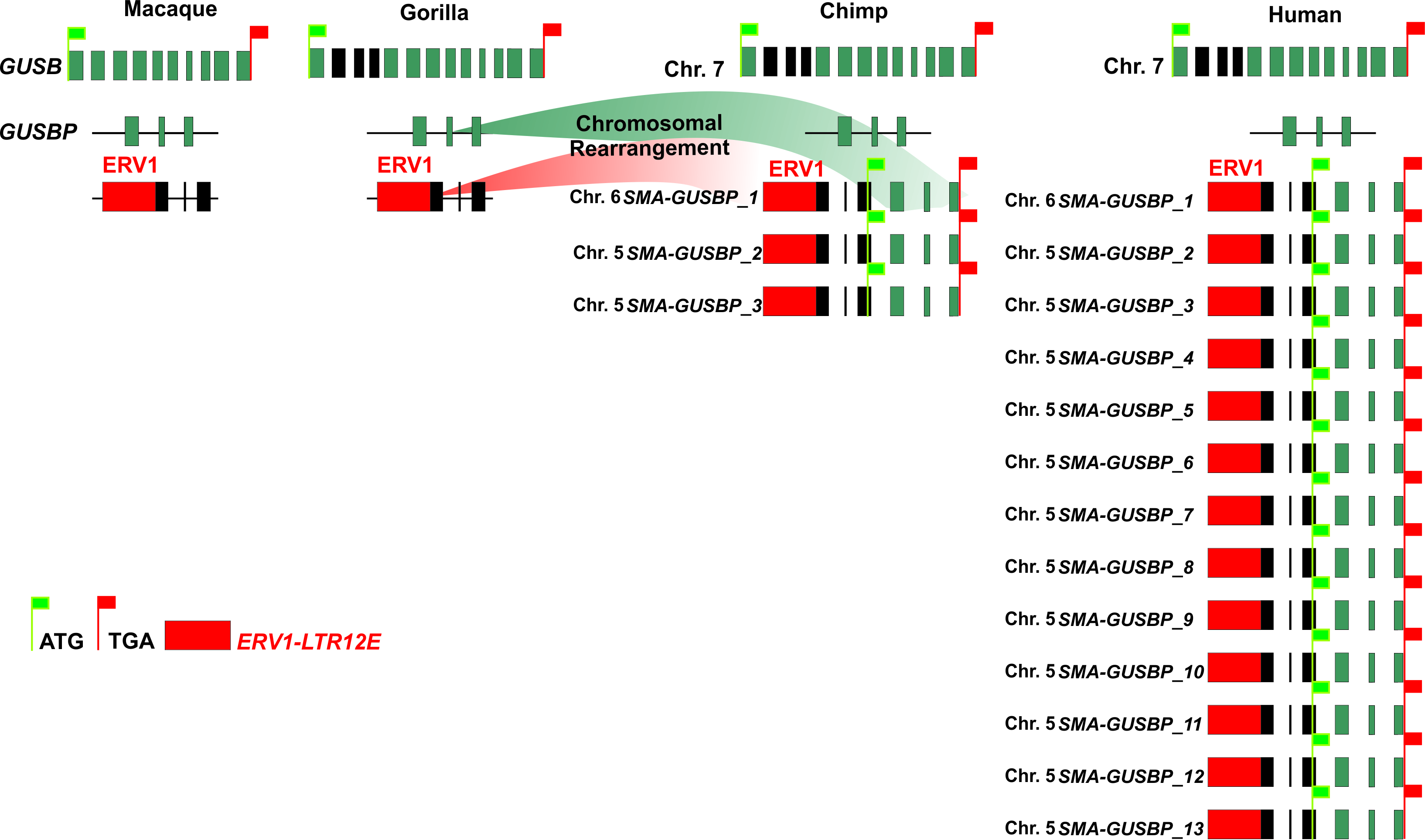


**Supplementary Figure 1. Evolution of the *SMA-GUSBP* gene family**

The structure, chromosomal location and copy numbers for the *SMA-GUSBP* gene duplicates were determined using the reference genomes from macaque, gorilla, chimpanzee (3 copies) and human (13 copies) (rheMac8, gorGor4, panTro4, hg38, respectively). Please note that the names of the *SMA-GUSBP* genes in humans do not agree with the UCSC genome browser annotations and need to be resolved upon detailed analysis.


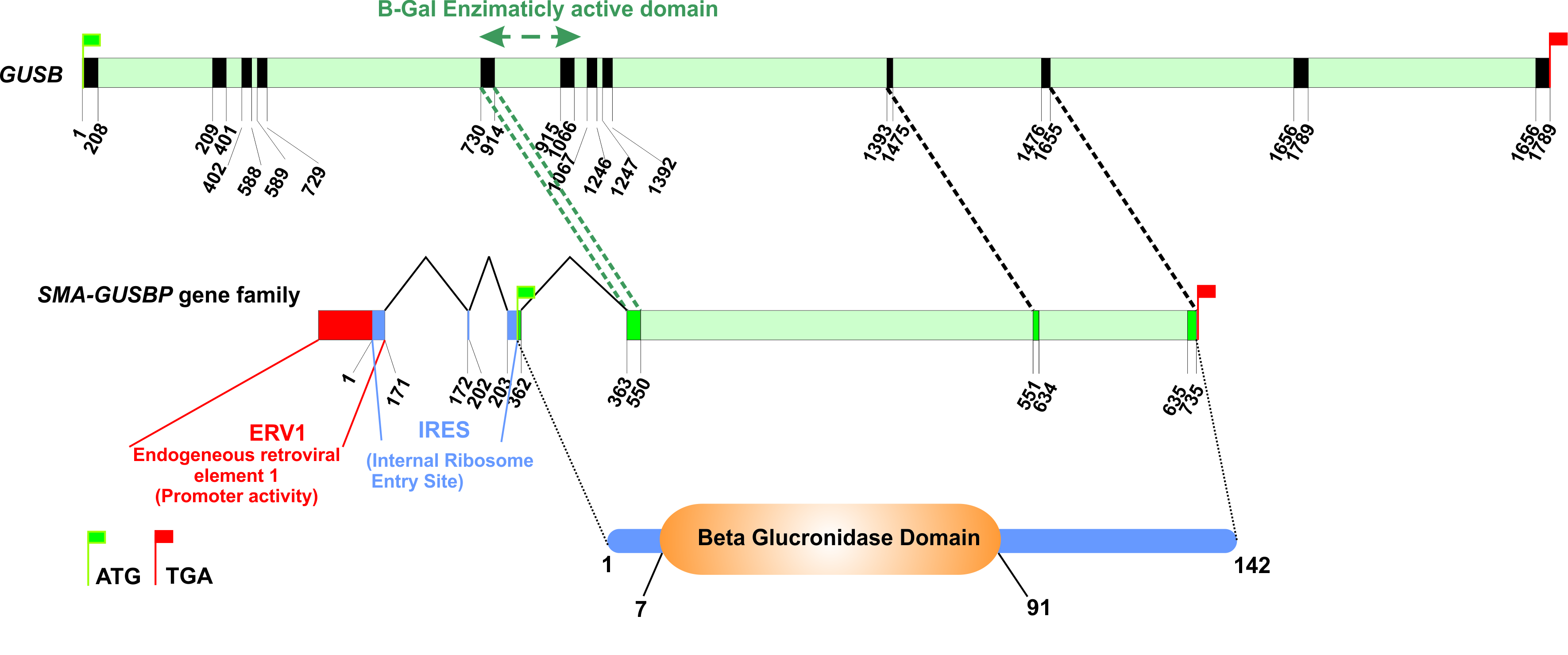


**Supplementary Figure 2. Gene and protein structure of *SMA-GUSBP***

The figure shows the mRNA structure of *SMA-GUSBP* (BC067351) aligned with human *GUSB* (NM_000181). The predicted protein structure of *SMA-GUSBP* is sketched at the bottom.

**
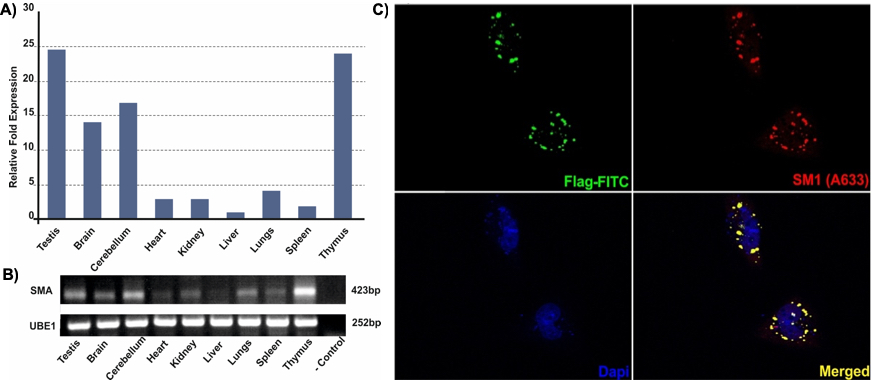
**

**Supplementary Figure 3. Expression and immunofluorescence analysis of flag-tagged SMA-GUSBP in HeLa cells**

**A)** Quantitative PCR analysis showing the relative expression of *SMA-GUSBP* genes in different human tissues. Expression data were first normalized against the housekeeping gene *UBE1* and then cross-compared using the liver as a reference. **B)** RT-PCR results from cDNA prepared from total RNA extracted from different tissues. *UBE1* expression is used as a positive control. **C)** Immunofluorescence analysis of HeLa cells over-expressing C-terminally FLAG-tagged *SMA-GUSBP3*. Cells were fixed with 4% paraformaldehyde 24 hours after transfection at room temperature for 20 min. The cells were stained with DAPI to detect the nuclei, the mouse monoclonal anti-FLAG antibody (green), and the polyclonal-rabbit antibody (SM1) (red) raised against SMA-GUSBP proteins.
